# High current gain transistor laser

**DOI:** 10.1038/srep27850

**Published:** 2016-06-10

**Authors:** Song Liang, Lijun Qiao, Hongliang Zhu, Wei Wang

**Affiliations:** 1 Key Laboratory of Semiconductor Material Sciences, Institute of Semiconductors, Chinese Academy of Sciences, Beijing, 100083, China

## Abstract

A transistor laser (TL), having the structure of a transistor with multi-quantum wells near its base region, bridges the functionality gap between lasers and transistors. However, light emission is produced at the expense of current gain for all the TLs reported up to now, leading to a very low current gain. We propose a novel design of TLs, which have an n-doped InP layer inserted in the emitter ridge. Numerical studies show that a current flow aperture for only holes can be formed in the center of the emitter ridge. As a result, the common emitter current gain can be as large as 143.3, which is over 15 times larger than that of a TL without the aperture. Besides, the effects of nonradiative recombination defects can be reduced greatly because the flow of holes is confined in the center region of the emitter ridge.

A transistor laser (TL)^1^,^2^,^3^, having the structure of a transistor with multi-quantum wells (MQWs) near its base region, bridges the functionality gap between lasers and transistors. From a TL, an electrical signal can be outputted simultaneously with a light signal by inputting one electrical signal, making it suitable for future high performance optoelectronic integrated device applications^4^. As a new kind of semiconductor laser or transistor, TLs have aroused many interests since its invention. For example, in 2006, the paper^2^ reporting the first room temperature operation of TLs was voted as one of the five most important papers published by Applied Physics Letters in over 40 years^5^. Because of the transistor structure, many interesting characters have been demonstrated, including resonance free frequency response, large direct modulation band width^6^, voltage controlled mode of operation^7^, low relative intensity noise (RIN) close to the shot-noise limit^8^ and low 3rd order intermodulation distortion (IMD)^9^.

However, light emission for all the TLs reported up to now is produced at the expense of current gain. Taking npn TLs as an example, in the devices, electrons injected from the emitter into the base layer first recombine with holes radiatively before the left being collected by the collector^4^. The majority of the electrons are consumed by stimulated light emissions, leading to a current gain which is a lot lower than the gain of a traditional transistor. The common emitter (CE) mode current gain (collector current/base current) is lower than 5 for most, if not all, of the TLs studied, either experimentally^1^,^2^,^3^,^6^,^7^,^8^,^9^,^10^ or numerically^11^,^12^,^13^. The low current gain may limit the performance of systems that use TLs. For example, it is much easier to integrate monolithically a heterojunction bipolar transistor (HBT) and a TL than to integrate an HBT with a laser diode (LD) because of the dual functionality of TLs. For such applications, a large current gain of TL (used as HBT) is desired for the amplification of electrical signal to drive the laser.

In this work, we propose a novel TL structure which has an n-doped InP layer inserted in the emitter ridge, forming a flow aperture in the center of the emitter ridge for only holes. Here after, the TLs having the hole current aperture is designated as a-TLs. The properties of the a-TLs are systematically studied numerically. It is shown that while the light emission power of a-TLs is comparable with that of TLs without the aperture at the same base current, the CE current gain of a-TLs can be over 15 times larger.

## Results

### Device structure

**Figure 1:**
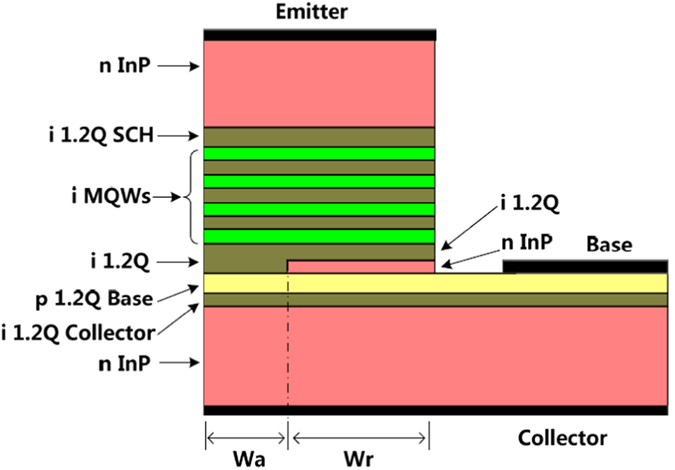
Schematic structure of the proposed a-TL. Only half the structure is shown.

Long wavelength devices based on InP material are used for the study. Figure 1 shows the structure of the proposed a-TL, which includes an n-InP substrate (1 × 10^18^ cm^−3^), a 50 nm undoped quaternary InGaAsP with a 1.2 μm emission wavelength (1.2Q) as a collector layer, a 100 nm p-doped 1.2Q base layer (5 × 10^18^ cm^−3^). In the outer parts of the emitter ridge, an n-doped InP layer is placed above the base layer. As will be shown below, the InP layer forms a current aperture in the center of the emitter ridge for only holes. As shown in Fig. 1, the width of the n-InP layer and the half width of the aperture are denoted as W_r_ and W_a_, respectively. A 30 nm undoped 1.2Q layer is inserted between the n-InP layer and the MQW layer. In the w_a_ region, all the material between the base layer and the MQWs is undoped 1.2Q. The top layer of the emitter ridge is a 1500 nm n-InP (1 × 10^18^ cm^−3^) layer. In the study, the default parameters of a-TLs are as following: The thickness and the doping level of the n-InP layer are 30 nm and 1 × 10^18^ cm^−3^, respectively. W_r_ = 2 μm and W_a_ = 1 μm. The separation between the edge of the base contact and the emitter ridge is 1.0 μm. The length of the device is 300 μm. The facet reflection of the device is set as 30 percent. All the simulations presented here are in the common emitter (CE) mode of TLs with a collector to emitter bias voltage (V_CE_) of 5 V. For comparison, the properties of deep ridge TLs^14^,^15^, where W_r_ = 0 μm, are also studied.

### High current gain of a-TLs

**Figure 2:**
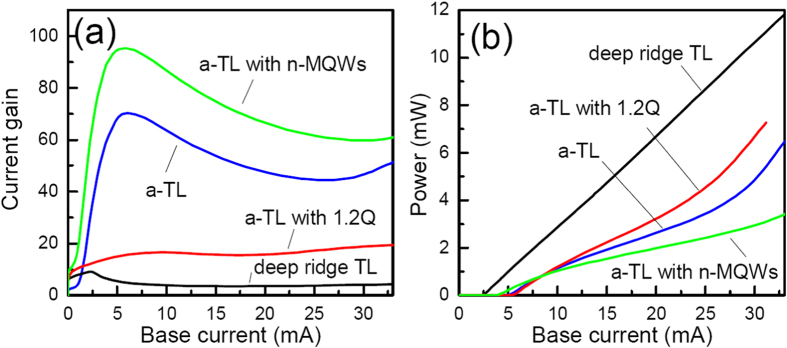
CE mode current gain (**a**) and light power (**b**) as a function of the base current for different types of TLs. In the figures, the a-TL line is for a device with default parameters, the a-TL with 1.2Q line is for a device, in which the n-InP layer is replaced by a n-1.2Q layer, the a-TL with n-MQWs line is for a device, in which the 1.2Q layer and MQWs above the n-InP layer are n-type doped with a 5 × 10^17^/cm^3^ concentration.

Figure 2(a,b) show the CE mode current gain and light power as a function of the base current for different types of TLs. Compared with a-TLs, the deep ridge TL has a relatively smaller threshold current, because of the better confinement of both the light and current in the device structure. The current gain of the deep ridge TL increases first to 9.3 at threshold current before decreasing to about 3.5 when the base current is further increased. Contrary to the low current gain of the deep ridge TL, as shown in Fig. 2(a), the highest current gain (at the threshold current of 6 mA) of the a-TL with the default parameters is 70.5, which is 7.5 times larger than that of the deep ridge TL. When the base current is in the range from 6 to 25 mA, the current gain of the a-TL keeps being larger than that of the deep ridge TL for over ten times at the same base current. At the same time, the light power difference between the two kinds of TLs is relatively quite small. For example, the light power of the a-TL at 33 mA base current is 6.4 mW, which is only 1.8 times smaller than that of the deep ridge TL at the same base current. It can be noted from Fig. 2(a) that there is gain compression after lasing for both the two kind of devices, which is typical for TLs and is resulted from the consumption of carriers by the laser emission^2^.

**Figure 3:**
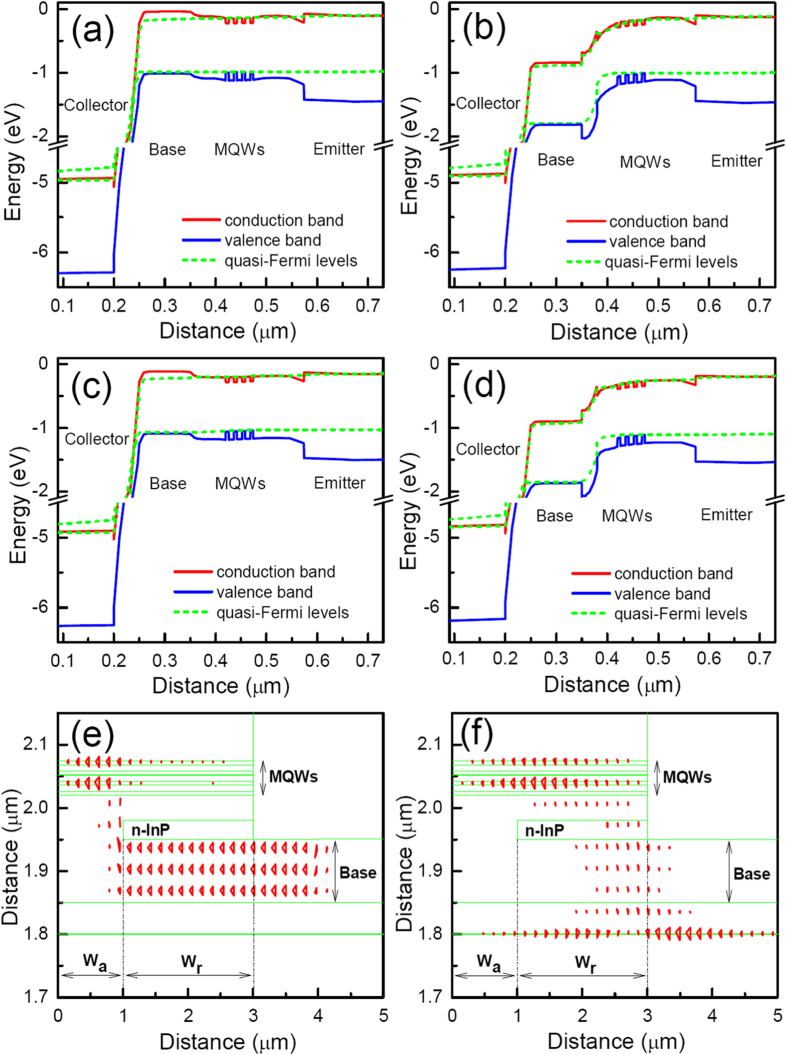
The band diagrams in W_a_ region (**a**) and W_r_ region (**b**), the cross-section hole current (**e**) and electron current (**f**) distributions of an a-TL with default parameters at 25 mA base current. (**c,d**) are band diagrams in W_a_ region and W_r_ region, respectively, of an a-TL, in which the 1.2Q layers and the MQWs above the n-InP layer are n type doped with a 5×10^17^/cm^3^ concentration.

The high current gain of the a-TL is resulted from the blocking of the flow of only holes between the base and the emitter by the n-InP layer. During laser operation of the a-TLs, the BE junction of the device is forward biased. The band diagram in the center (W_a_) region of the a-TL, where there is no n-InP is shown in Fig. 3(a). As can be seen, the band diagram above the collector is similar to the diagram of a normal diode laser. Laser emission is produced by the recombination of holes injected from the base layer with the electrons from the emitter layer in the MQWs. The band diagram of the a-TL in the W_r_ region of the emitter ridge, however, is noticeably different because of the presence of the n-InP layer. As seen from Fig. 3(b), the upper part of the n-InP layer and part of the undoped 1.2Q material above the n-InP layer is depleted. Electrons in the MQWs injected from the emitter diffuse to the depleted region and then dragged by the electric field in the region toward the base direction before they are injected into the base layer. The flow of holes from the base layer to the MQWs, however, is blocked effectively by the energy barrier in the valance band between the 1.2Q base and the n-InP layer.

The cross-section hole and electron current distributions are shown in Fig. 3(e,f), respectively. As can be seen, while the electrons can flow from the emitter to the base in both the W_a_ and W_r_ regions, the hole current flow is restricted in only the W_a_ region, forming a current aperture for only the holes in the center of the emitter ridge with the help of the n-InP layer. The majority of holes that flow through the aperture recombine radiatively with electrons in the MQWs. In the W_r_ regions of the emitter, the fraction of electrons consumed by radiative recombination is much smaller than that in the W_a_ region because of the lack of holes. Under the same BE bias voltage, the number of electrons injected from the emitter into the base increases noticeably relative to the case of deep ridge TL, leading to the increase of the current gain. As more electrons are present in the base region, more of them diffuse to the base contact, increasing the portion of electron current in the total base current^11^. Because the electron current does not contribute to the laser emission, the slope efficiency of the a-TL is lower than that of the deep ridge TL and is varied with the base current as shown in Fig. 2(b).

### Effects of device parameters on the properties of a-TLs

The energy barrier in the valance band, which is formed because of the band discontinuity between InP and 1.2Q material, is crucial for the high gain of the a-TL. An a-TL device with all the same default parameters but the n-InP is replaced by n-1.2Q is also studied. As shown in Fig. 2, while there is only a minor change in light power, the current gain is reduced noticeably from 70 to 15 at 5 mA base current. In an actual device, the 1.2Q and MQW layers above the n-InP layer may exhibit n-type doping even without n-type impurity added intentionally. The band diagrams in the W_a_ and W_r_ regions of an a-TL having n-type doping in the layers, whose level is as high as 5×10^17^/cm^3^, are shown in Fig. 3(c,d), respectively. As can be seen, because of the doping the conduction band spike between the n-InP layer and the 1.2Q layer is increased as compared to when there is no doping. In spite of this higher spike, the current gain is increased, which is 95 at 5 mA base current, as shown in Fig. 2(a). N-type doping in the 1.2Q and MQW layers increases the number of electrons that can be collected by the base layer, which counteracts the effects of the higher spike.

**Figure 4:**
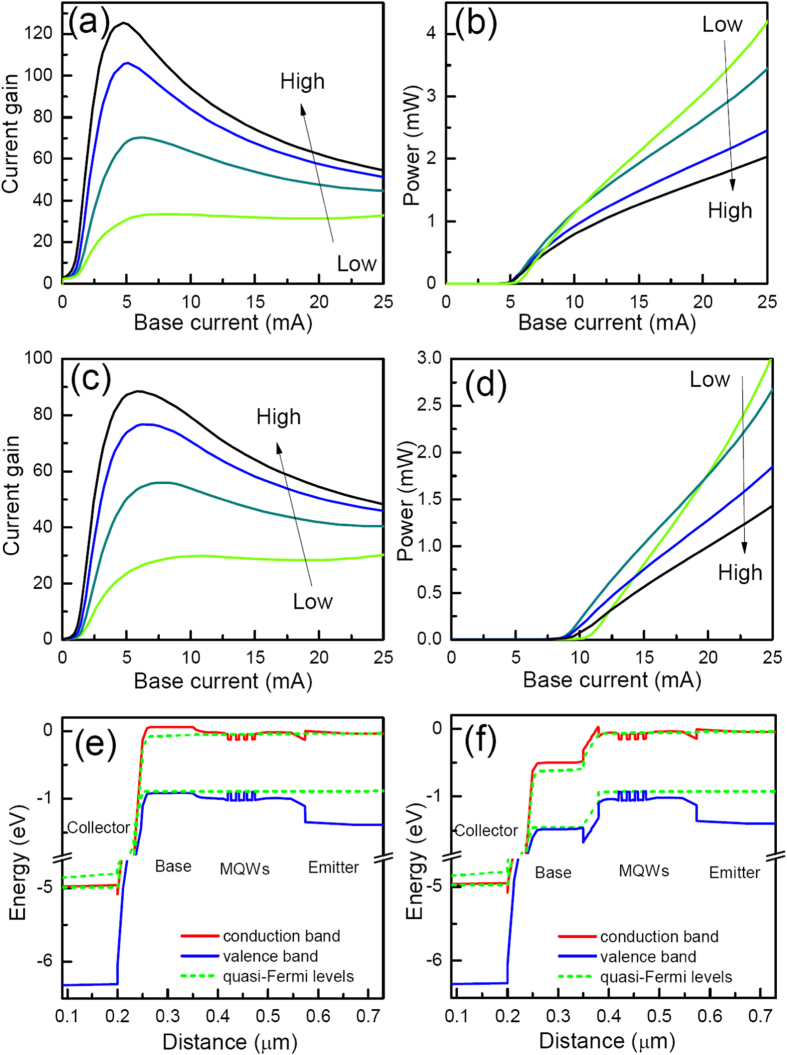
The current gain (**a,c**) and light power (**b,d**) characteristics of a-TLs having n-InP layers with different doping concentrations, which are 0.1, 1, 2, and 3 × 10^18^/cm^3^, respectively. The band diagrams in W_a_ and W_r_ region of the a-TL with 0.1 × 10^18^/cm^3^ doping in the n-InP layer are shown in (**e,f**). (**a,b,e,f**) are of TLs with no defects. (**c,d**) are of TLs with 1 × 10^6^ cm•s^−1^ surface recombination velocity on the MQW side walls.

The current gain and light power characteristics of a-TLs having n-InP layers with different doping concentrations are shown in Fig. 4(a,b), respectively. As can be seen, the current gain of the device at 5 mA base current is increased significantly from 3.5 to 125.2 when the doping level of the n-InP layer is varied from 0.1 to 3 × 10^18^/cm^3^. The band diagrams in the W_a_ and W_r_ regions of the emitter ridge of the a-TL having n-InP layer with 0.1 × 10^18^/cm^3^ doping are shown in Fig. 4(e,f), respectively. The diagram in the W_a_ region is similar to that of the a-TL with n-InP layer having 1 × 10^18^ cm^−3^ doping concentration. In contrast, the diagram in the W_r_ region is different because only the InP layer is depleted. Besides the energy barrier in the valance band, there is also an energy barrier in the conduction band as shown in Fig. 4(f). Thus, the flow of both electrons and holes between the base and the emitter is blocked, leading to the decrease of current gain with the decrease of the doping level in the n-InP layer. A higher current gain corresponds to a larger amount of electrons injected from the emitter into the base layer, which will increase the number of electrons collected by the base contact, leading to the decrease of the slope efficiency of the light emission with the doping level as can be seen from Fig. 4(b).

**Figure 5:**
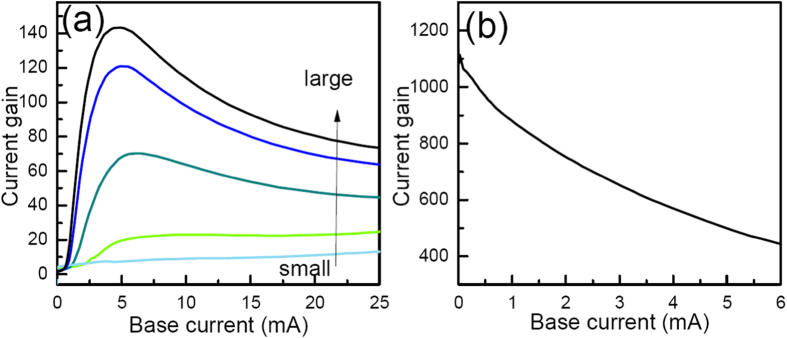
(**a**) The effects of W_r_ on the current gain of a-TLs, W_r_ in the figure are 0.5, 1.0, 2.0, 3.0 and 3.5 μm, respectively. (**b**) current gain of the a-TL with W_a_ = 0.

As shown in Fig. 5(a), when W_r_ is increased from 0.5 μm to 3.5 μm, the current gain at 5 mA base current is increased from 7.7 to 143.3, which is over 15 times larger than the largest gain of the deep ridge TL. The light power of the a-TL with W_r_ = 3.5 is 2.8 mW at 25 mA base current. The slope efficiency (not shown here) of the light emission decreases with the current gain, which is similar to the trend shown in Fig. 4. For applications such as monolithic integration of a laser with transistors, light emission may not be needed at all when an a-TL is used as a transistor for current amplification. Thus W_r_ can be set as 0 μm. In such a case, the current gain of the device can be over 500 at 5 mA base current as shown in Fig. 5(b), which helps to obtain high performance integrated devices. The thickness of the n-InP layer is also varied from 10 nm to 40 nm. At 5 mA base current, the current gain increases with the thickness from 54.6 to 70 when it is smaller than 30 nm. No apparent change of current gain is observed as the thickness of the InP layer is further increased to 40 nm.

### Properties of a-TLs with defects on the emitter side walls

When there are defects at the side walls of the emitter ridge, the properties of the deep ridge TLs deteriorate seriously because of the consumptions of carriers by the nonradiative recombinations^16^. To account for the nonradiative recombination centers, the surface recombination velocity at the surface of the exposed MQWs is set as 1 × 10^6^ cm•s^−1^ for a surface, for example, just after dry etching. Our simulations show that the threshold current of the deep ridge TL increases significantly to be over 1000 mA and the current gain decreases to be lower than 0.03 at around 5 mA base current. As can be seen from Fig. 4(c,d), another interesting point of a-TLs is that the effects of the nonradiative recombinations at the defects can be decreased greatly. Even with this very high the surface recombination velocity, the threshold currents of the a-TLs are only about 10 mA. In the a-TLs, because the hole flow is confined in the W_a_ region in the center of the emitter ridge, most of the holes combine with electrons radiatively before diffusing to the defects which is 2 μm away. Thus the effects of the defects can be well screened.

## Discussion

In the a-TLs as shown in Fig. 1, the MQWs are placed above the p-doped base layer. Thus both the diffusion of p-type dopant (Zn is usually used for InP material system) into the MQWs and the optical absorption of the p-type base material can be reduced, helping to improve the optical properties of the device^11^. Only a simple two step epitaxy process is needed to fabricate a-TLs. The material layers from the buffer layer to the n-InP layer are successively grown in the first epitaxy run. Then an opening with a width of 2W_a_ is formed in the n-InP layer. The property tunings as shown in Fig. 5 can be realized easily by simply varying the parameters of mask patterns for photolithography. In the fabrication process of the InP based TLs as in Fig. 1, when H_2_SO_4_:H_2_O_2_:H_2_O is used for the etching of InGaAsP material and HCl:H_2_O is used for the etching of InP, the emitter ridge can be terminated precisely above the base layer. Thus a base layer having as small as one to several tens of nanometer thickness, which is essential for ultra high speed operation of transistors, can be adopted. However, it is difficult to have a base layer this thin for other kinds of TLs, because of constrains from either the structure of the base layer for a shallow ridge TL^2^ or from the fabrication process of a deep ridge TL^11^. Finally, it is worth noting that though the device structure studied in this work is ridge waveguide npn-type, the proposed structure can be used in other types of TL such as pnp TLs and vertical cavity TLs^17^.

In summary, we study numerically a novel design of TLs, which has an n-doped InP layer inserted in the emitter ridge. While electrons can flow across the n-InP layer from the emitter to the base, the flow of holes from the base to the emitter can be blocked by the n-InP layer, forming a hole current path in the center of the emitter ridge. By varying the parameters of the n-InP layer, the CE mode current gain can be as large as 143.3, which is over 15 times larger than that of the deep ridge TL. Besides, the effects of nonradiative recombination defects at the emitter side walls can be reduced greatly.

## Methods

### Device modeling

The simulation models of the device are developed by Crosslight PICS 3D software. The related physical models can be found in ref. 18. PICS3D numerically solves the electrical, optical, MQW gain and laser rate-equation models self-consistently based on the finite-element-method. The classic drift-diffusion model is used to describe the carrier transport^18^, with the thermionic-emission model used at the heterojunctions. Lateral optical modes are calculated by the effective-index method^18^. The gain calculations are based on 4 × 4 kp band method, including valence-mixing effects. A Lorentz broadening function is used with 0.1 ps scattering time.

### MQW parameters

The MQWs of the device have a 1.5 μm emission wavelength and consist 4 compressively strained QWs and 4 unstrained 1.2Q barriers with thicknesses of 5 nm and 10 nm, respectively. An 80 nm undoped 1.2Q layer is used as upper separate confinement heterostructure (SCH) layer of the MQW active layer.

## Additional Information

**How to cite this article**: Liang, S. *et al*. High current gain transistor laser. *Sci. Rep.* **6**, 27850; doi: 10.1038/srep27850 (2016).

## References

[b1] Walter G., Holonyak N. Jr., Feng M. & Chan R. Laser operation of a heterojunction bipolar light-emitting transistor. Appl. Phys. Lett. 85, 4768–4770 (2004).

[b2] Feng M., Holonyak N. Jr., Walte r G. & Chan R. Room temperature continuous wave operation of a heterojunction bipolar transistor laser. Appl. Phys. Lett. 87, 131103 (2005).

[b3] Dixon F. . Transistor laser with emission wavelength at 1544 nm. Appl. Phys. Lett. 93, 021111 (2008).

[b4] Holonyak N. & Feng M. The transistor laser. IEEE Spectrum 43, 50–55 (2006).

[b5] Kloeppel J. E. Illinois researchers produce two most important scientific papers, Eurekalert, http://www.eurekalert.org/pub_releases/2006-06/uoia-irp060506.php (2006), date of access 5 June 2006.

[b6] Tan F., Bambery R., Feng M. & Holonyak N. Jr. Transistor laser with simultaneous electrical and optical output at 20 and 40 Gb/s data rate modulation. Appl. Phys. Lett. 99, 061105 (2011).

[b7] Feng M., Holonyak N. Jr., Then H. W., Wu C. H. & Walter G. Tunnel junction transistor laser. Appl. Phys. Lett. 94, 041118 (2009).

[b8] Tan F., Bambery R., Feng M. & Holonyak N. Jr. Relative intensity noise of a quantum well transistor laser. Appl. Phys. Lett. 101, 151118 (2012).

[b9] Then H. W., Tan F., Feng M. & Holonyak N. Jr. Transistor laser optical and electrical linearity enhancement with collector current feedback. Appl. Phys. Lett. 100, 221104 (2012).

[b10] Sato N. . Design and characterization of AlGaInAs/InP buried heterostructure transistor lasers emitting at 1.3-μm wavelength. IEEE J. Sel. Top. Quantum Electron. 19(4), 1502608 (2013).

[b11] Liang S. . Temperature performance of the edge emitting transistor laser. Appl. Phys. Lett. 99, 013503 (2011).

[b12] Duan Z. G. . Design and epitaxy of 1.5 μm InGaAsP-InP MQW material for a transistor laser. Opt. Express 18, 1501–1509 (2010). 20173978 10.1364/OE.18.001501

[b13] Shi W., Chrostowski L. & Faraji B. Numerical study of the optical saturation and voltage control of a transistor vertical-cavity surface-emitting laser. IEEE Photon. Technol. Lett. 20, 2141–2143 (2008).

[b14] Liang S. . InP-based deep-ridge NPN transistor laser. Opt. Lett. 36, 3206–3208 (2011). 21847209 10.1364/OL.36.003206

[b15] Qiao L. J. . Continuous-wave operation up to 20 °C of deep ridge npn InGaAsP/InP multiple quantum well transistor laser emitting at 1.5 μm wavelength. Opt. Express 23, 11388–11393 (2015). 25969233 10.1364/OE.23.011388

[b16] Huo W. J. . Fabrication and characterization of deep ridge InGaAsP/InP light emitting transistors. Opt. Express 22, 1806–1814 (2014). 24515189 10.1364/OE.22.001806

[b17] Wu M. K., Feng M. & Holonyak N. Jr. Surface emission vertical cavity transistor. IEEE Photon. Technol. Lett. 24, 1346–1348 (2012).

[b18] Crosslight Device Simulation Software General Manual, version 2011, Crosslight Software Inc. (2011).

